# Prevalence and Determinants of Wheezing and Bronchodilatation in Children With Cystic Fibrosis: A Retrospective Cohort Study

**DOI:** 10.3389/fped.2022.856840

**Published:** 2022-05-12

**Authors:** Francois Galodé, O. Ladipo, A. Andrieux, H. Feghali, S. Bui, Michael Fayon

**Affiliations:** ^1^Paediatric Cystic Fibrosis Reference Center, Hôpital Pellegrin-Enfants, CHU de Bordeaux, Bordeaux, France; ^2^Service de Pédiatrie, CHU de la Mère et de l’Enfant Lagune, Cotonou, Benin; ^3^INSERM, Centre d’Investigation Clinique (CIC 1401), University of Bordeaux, Bordeaux, France

**Keywords:** asthma, wheezing, bronchial hyperresponsiveness, cystic fibrosis, respiratory function tests

## Abstract

**Background:**

Many patients with cystic fibrosis (CF) wheeze, and are dubbed as having CF-asthma. Understanding the determinants of such wheezing may avoid unnecessary treatments and open newer treatment avenues.

**Objectives:**

Main: To evaluate the prevalence and characteristics of wheezing and a positive bronchodilatory response (BDR) in children with CF. Secondary: To identify the predictive markers and the impact of current wheezing a positive BDR.

**Methods:**

A retrospective single-center study in children with CF. We determined the characteristics of physician-reported wheeze in patients <6 years, and a BDR in patients aged 6–17 years. Anthropometric, lung function, laboratory, genetic and microbiological data were recorded in all groups. Variables were compared using the Chi^2^ and Student *t*-tests, and ANOVA.

**Results:**

125 preschool and 69 school-aged children and adolescents with CF were included in the study. 71.2% of patients <6 years of age had had at least one episode of wheezing: 26.3% of patients were Transient Early Wheezers, 12.6% Late Onset Wheezers and 37.9% were Persistent Wheezers. The prevalence of a positive BDR was 73.5, 48.5, and 52.9% in the 6–8 years, 10–12 years, and 15–17 years age groups, respectively. Allergic factors were not predictive of wheezing in preschoolers. In the 6–8 years age group, the sum of wheal diameters of allergic skin prick tests (SPT, house dust mite + cat + dog dander) was greater in those with a BDR vs. no BDR (4 [2.0–8.8] vs. 1 [0–7.0] mm, *p* = 0.01). The presence of *Pseudomonas aeruginosa* in the bronchial secretions before 3 years of age was not significantly associated with either the presence of wheezing at the age of 6 years or a BDR in school-aged children and adolescents. The proportion of homozygous p.F508del patients was significantly lower in the group of patients who had wheezed by 6 years of age (60% vs. 72.7%, *p* = 0.009), but higher in the 6–8 years old group with a BDR vs. no BDR (64% vs. 36%, *p* = 0.04). Current wheezers at 6 years had a lower mean FEV_1_ vs. the non-current wheezers (91.5 ± 4.4% vs. 100.9 ± 2.4%; *p* = 0.047). Similarly, forced vital capacity (FVC) was significantly lower in the 6–8 years old group with BDR vs. no BDR (85 ± 19 vs. 101 ± 21%, *p* = 0.015).

**Conclusion:**

Wheezing and BDR are very frequent findings in children with CF. Current wheeze at the age of 6 years was associated with worse lung function. Labeling wheezing in CF as “CF-Asthma” is misleading since the determinants are different, and may lead to inappropriate prescriptions of inhaled steroids.

## Introduction

Cystic fibrosis (CF) is one of the most frequent and severe genetic diseases affecting European populations. Clinical manifestations can vary from mild to severe forms, and prognosis depends to a large extent on the severity of respiratory impairment. This associates chronic bronchial inflammation and infection, and the presence of very thick mucus, a consequence of the absence or dysfunction of the CFTR channels present on the apical surface of bronchial epithelial cells. Bronchiectasis will ensue, followed by chronic respiratory failure.

Asthma and CF are both chronic inflammatory diseases of the lung, but their pathophysiology is quite different. Airway inflammation is mostly eosinophilic in allergic asthma, whereas it is predominantly neutrophilic in CF (1, 2). Wheezing is very common in asthma. A prevalence of 30–50% has been reported in preschool children, in whom less than half will undergo persistent symptoms (1). Wheezing, whether in patients with asthma or CF, is classically the consequence of airway obstruction due to inflammation, bronchospasm, non-discharged secretions and/or small airways.

Morphologically, the bronchi of patients with asthma and CF share common features, such as the development of bronchial remodeling, including hyperplasia of the bronchial smooth muscle (3, 4). Bronchial hyperresponsiveness (BHR) is also observed in both conditions. This is usually demonstrated by the onset of bronchoconstriction induced by a specific or non-specific stimulus, and/or reversibility after exposure to a bronchodilator (5). Up to half of all patients with CF have measurable BHR, which does not appear to be related to the patient’s atopic status (6–8). According to Weinberger BHR in CF shows characteristics which are different from asthma, e.g., bronchodilation on exertion (9).

Alterations in the signaling pathways regulating airway smooth muscle (ASM) contractility in CFTR-deficient patients have been described. As an example, persistent bacterial infection, especially by *Pseudomonas aeruginosa*, stimulates the release of interleukin-8 from the airway epithelium, resulting in neutrophilic inflammation. Increased neutrophilia and CFTR-deficient T-helper cells create an inflammatory environment characterized by high levels of Tumor Necrosis Factor (TNF). The presence of high levels of TNFα, Interleukin-8 and Interleukin-13, may contribute to increased ASM contractility which participates in wheezing and BHR (10).

Predictive indices and scores have been proposed to identify which of the early non-CF wheezing children are at risk of persistence (of wheezing) at school age. Two of these have been validated in different populations: the PIAMA score (11, 12) and the Asthma Predictive Index (API) (13). A positive API index indicates a 2.6–9.8 greater risk of current asthma during school age (6 and 13 years) (14). According to the PIAMA score the risk of asthma from age 6 to 8 years is as follows: <5% (score 0–7); 6–22% (score 8–15); 25–60% (score 16–23) (12).

There is a lack of such data in children with CF. Our primary objective was therefore to evaluate the prevalence and characteristics of wheezing and a bronchodilatory response (BDR) to short-acting beta-agonists as a marker of airway lability in a population of children with CF followed in a single large CF center. The secondary objectives were to identify the factors associated with wheezing and a positive BDR, and to determine the impact of current wheezing at the age of 6 years.

## Materials and Methods

We conducted a retrospective, analytic, single-center study in children with CF, aged less than 18 years, followed at the Regional Pediatric CF-center in Bordeaux University Hospital, France. In this retrospective study French regulatory legislation requires that the protection of personal data is ensured, and this was applied in our study (15). The Bordeaux University Hospital Institutional Research Ethics Board authorized the conduct and publication of this research (Reference CERBDX-2022-03).

The ***first part*** of the study involved children aged less than 6 years, recruited from February 2016 to June 2016. The primary objective was to determine the overall frequency of wheezing in such children. The secondary objectives were to describe the characteristics of wheezing according to the TUCSON clinical phenotypes as described by Martinez et al. (16), to determine predictive markers for the persistence of wheezing at school age (6 years), and to describe the consequences of wheezing particularly with respect to lung function at 6 years. Children were assigned to four categories according to their history of wheezing: those who had no recorded lower respiratory tract illness with wheezing during the first 3 years of life and had no wheezing at 6 years of age (NW); those with at least one lower respiratory tract illness with wheezing during the first 3 years of life but no wheezing at 6 years of age (those with transient early wheezing, TW); those who had no lower respiratory tract illness with wheezing during the first 3 years of life but who had wheezing at 6 years of age (those with wheezing of late onset, LW); and those who had at least one lower respiratory tract illness with wheezing in the first 3 years of life and had wheezing at 6 years of age (those with persistent wheezing, PW) (16).

Data collected over the first 6 years of life were the following: Demographic (age, weight, and gestational age), family (asthma and atopy, *in utero* and postnatal cigarette smoke exposure), respiratory function (best lung function (LFT) results between age 6 and 7 years), immunological (allergic Skin Prick Tests and total IgE or specific IgE to the most common respiratory allergens: (house dust mite, cat or dog dander)), bacteriological (pathogens found in the sputum) and therapeutic (treatments prescribed for respiratory symptoms. From these data, we scored the Asthma Predictive Index, the PIAMA score and determined the “TUCSON” wheezing phenotypes (16).

The ***second part*** of the study involved children aged 6–17 years from 2002 to 2011 and focused on the presence of a BDR. The preschool children shown in the above section were not included in this >6 years age school age study group. Three age ranges were selected: ≥6 years to <8 years, ≥10 years to <12 years, and ≥15 years to <17 years. For each patient, anthropometric, clinical, allergic (total IgE, blood eosinophils, and allergic skin tests), bacteriological, genetic, and spirometric data were collected. LFT criteria regarding bronchial obstruction and the BDR were reported according to the ATS/ERS recommendations. The criteria for a positive BDR were: improvement in ppFEV1 by at least 12%, of ppFEF_25–75_ or ppFEV1 by at least 35%; decrease in ppRV (and/or ppRV/TLC) by at least 20%, decrease in R_*aw*_ by at least 35%. The BDR was analyzed either based solely on ppFEV1 criteria, or on all the above-mentioned criteria (positive BDR if at least one of the functional reversibility parameter was present).

Current wheezing at 6 years of age was defined as children who had presented at least one episode of wheezing in the previous 12 months.

Regarding continuous variables, data are presented as medians and interquartile ranges, or means and standard deviations, according to their distribution. The statistical analysis was performed using NCSS software (Kaysville, Utah). The qualitative variables were compared using the Chi^2^ test. Quantitative variables were compared using a Student’s *t*-test if two groups were studied and by analysis of variance (ANOVA) in the presence of >2 groups (with a Bonferroni test to determine statistical differences between groups). A *p*-value < 0.05 was considered statistically significant.

## Results

One hundred and twenty-five patients aged less than 6 years of age and 69 patients above 6 years of age were included in the study.

### Preschool Children (<6 Years)

#### Demographic Data

Mean age of patients at the time of the retrospective data collection was 9.9 ± 4.9 years. The M:F sex ratio was 1:1.2 [girls: 69/125 (55.2%)]. These patients were full-term babies in 92% of cases, exposed to cigarette smoke *in utero* and postnatally in 25.6% and 59.5% of cases, respectively, and had at least one asthmatic parent in 20.3% (25/123) of cases. The p.Phe508del mutation was present in 87% (110/126) of cases (Table 1).

**TABLE 1:** Population characteristics according to the TUCSON phenotypes in preschool children aged less than 6 years.

	Total	NW	TW	LW	PW
***N***	125	22	25	12	36
Gender (G/F)	56/69	10/12	10/15	6/6	13/23
Age (years)	9.9 ± 4.9				
**Genotypes**					
Homozygous p.Phe508del	44 (55/125)	72.7 (16/22)*^#/##^*	32 (8/25)^##^	53 (7/12)	41.7 (15/36)^#^
Heterozygous p.Phe508del	45.6 (57/125)	27.3 (6/22)	48 (12/25)	41.7 (5/12)	44.4 (16/36)
Others	10.4 (13/125)	0 (0/22)	20 (5/25)	0 (0/12)	13.9 (5/36)
Birth weight (g)	3,158 ± 587	3,070 ± 126	3,228 ± 118	2,942 ± 171	3,131 ± 99
Full-term birth	92 (115/124)	95.4 (21/22)	92.0 (23/25)	91.6 (11/12)	91.6 (33/36)
≥1 parent with asthma	20.3 (25/123)	27.3 (6/22)	16.6 (4/24)	16.6 (2/12)	28.6 (10/35)
**Smoking**					
*in utero*	25.6 (31/121)	14.3 (3/21)	33.3 (8/24)	16.6 (2/12)	35.3 (12/34)
Postnatal	59.5 (72/121)	61.9 (13/21)	66.6 (16/24)	58.3 (7/12)	50 (17/34)
Personal allergy before age 6 years*					
Eczema	20 (24/120)	9.1 (2/22)	34.8 (8/23)	25.0 (3/12)	18.2 (6/33)
Rhinitis	9.2 (11/120)				
**Respiratory sensitization before age 6**					
SPT positive** House Dust Mite	6.7 (5/74)				
Cat	4.0 (3/75)				
Dog	2.7 (2/73)				
Sum specific IgE (HDM+Cat+Dog) (kUI/l)	2.0 [0.5–134.9]				
**Food sensitization before age 6 years**					
SPT** and/or RAST*** positive	23.4 (29/124)	18.6 (4/22)	20.8 (5/24)	16.7 (2/12)	33.3 (12/36)
Positive API at age 3 years	80.4 (41/51)	0	83.3 (10/12)	80.0 (8/10)	76.2 (16/21)
BMI *z*-score at age 3 years	−0.39 ± 0.09	−0.38 ± 0.19	−0.40 ± 0.17	−0.90 ± 0.25	−0.19 ± 0.16
**Bacteriology**					
*S. aureus* 0–3 years	87.7 (93/106)	89.5 (17/19)	90.0 (18/20)	88.9 (8/9)	86.2 (25/29)
*P. aeruginosa* 0–3 years	42.6 (46/108)	55 (11/20)	52.4 (11/21)	22.2 (2/9)	44.8 (13/29)
*P. aeruginosa* (chronic) 0–3 years	17.6 (19/108)	25.0 (5/20)	23.3 (5/21)	0 (0/9)	10.3 (3/29)

*n/N; mean ± standard deviation; % (n/N); median [min, max].*

*Reported by parents.

**SPT positive if papule >3 mm.

***RAST positive if >0.1 kUI/l.

*NW, never wheezers; TW, transient wheezers; LW, late wheezers; PW, persistent wheezers.*

*#NW vs. PW (p = 0.037); ##NW vs. TW (p = 0.009).*

#### Prevalence of Wheezing

**FIGURE 1:**
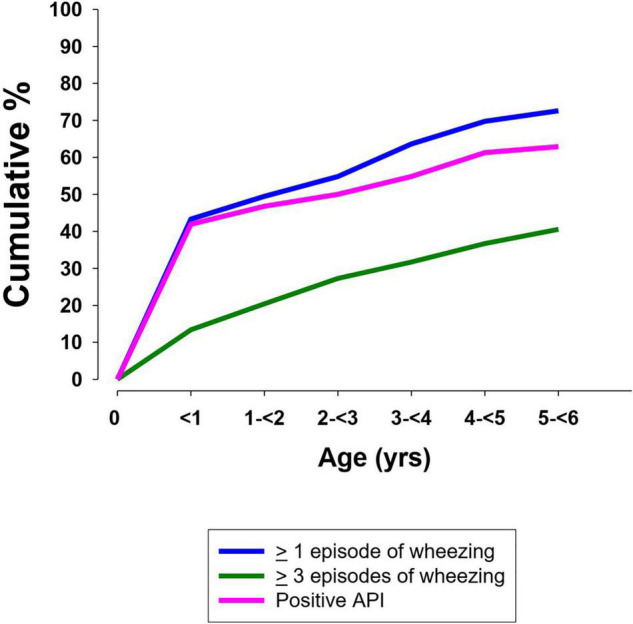
Cumulative frequency of wheezing and a positive Asthma Predictive Index in preschool children (<6 years) with CF (13).

The cumulative yearly frequency wheezing is shown in Figure 1. At least one episode of wheezing during the first 6 years of life was recorded in 71.2% (89/125) of cases. Regarding 115 children who had attained the age of 3 years, 66.1% (76/115) had had at least one episode of wheezing. In patients reaching the age of 6 years the cumulative prevalence of children having wheezed at least once was 76.8% (73/95) and 48.4% (46/95) had wheezed at least three times.

#### Prognostic Scores

**FIGURE 2:**
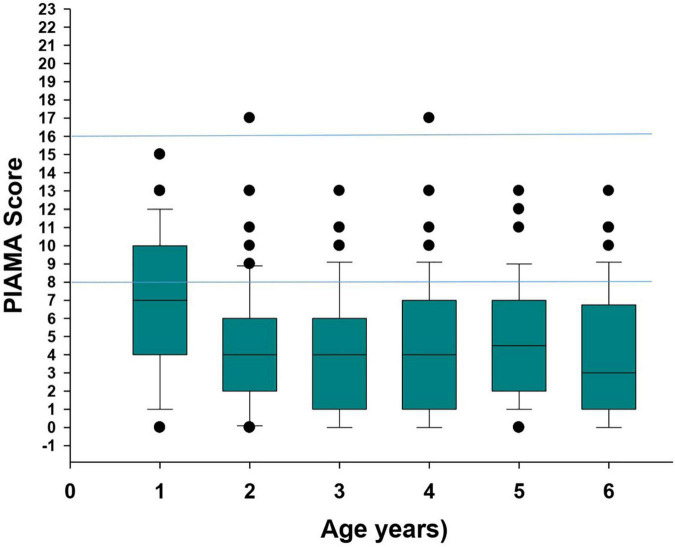
PIAMA* score in children with CF. Box and whisker indicating the median (horizontal line) and the upper and lower quartiles of the PIAMA* score. The black dots indicate the outliers. The blue horizontal lines indicate the risk of asthma in non-CF patients between 6 and 8 years of age according to the PIAMA* score: 0–7: low; 8–15: medium; 16–23: high. *PIAMA, prevention and incidence of asthma and mite allergy (12).

The API index was available in 72 of 73 children who had wheezed at least once from birth to the age of 6 years. A positive API between 0 and 3 years of age was present in 77.3% (34/44) of cases. The yearly cumulative frequency of a positive API is shown in Figure 1. Figure 2 indicates the PIAMA risk score.

#### Phenotypes

**FIGURE 3:**
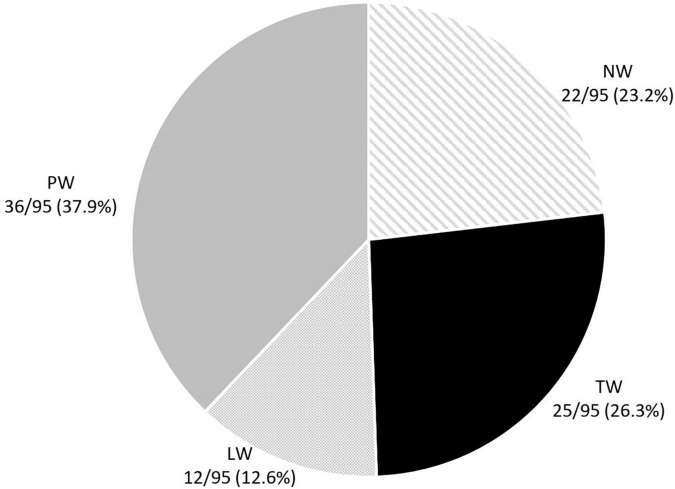
Distribution of the preschool wheezing phenotypes according to the TUCSON* phenotypes. *Tucson phenotypes: TW, transient earlier wheezers; NW, never wheezers; LW, late onset wheezers; PW, persistent wheezers (16).

Twenty-two (23.2%) children had never wheezed by 6 years of age (NW). The distribution of 73 children with wheezing was as follows: 25 (26.3%) TW, 12 (12.6%) LW, and 36 (37.9%) PW (Figure 3).

#### Predictive Markers

These are shown in Table 2. Homozygous p.Phe508del mutations were present in 72.7% of the children in the NW group compared to 32% in the TW group (*p* = 0.009) and 41.7% in the PW group (*p* = 0.037).

**TABLE 2:** Consequences of pre-school wheezing according to the TUCSON phenotypes.

Data	Total	NW	TW	LW	PW
**BMI**					
6 years (*z*-score)	0.08 ± 0.11	−0.18 ± 0.22	−0.28 ± 0.20	−0.57 ± 0.33	0.28 ± 0.18
**ppFEV_1_**					
6–7 years % theoretical value	100.4 ± 1.9	**107.8 ± 3.8**	98.4 ± 4.0	97.7 ± 5.0	97.9 ± 3.0*
**ppFVC**					
6–7 years % theoretical value	98.4 ± 1.9	105.9 ± 4.9	97.6 ± 3.9	91.2 ± 4.9	96.7 ± 2.9
**ppFEF_25–75_**					
6–7 years % theoretical value	87.0 ± 2.9	**99.6 ± 5.6**	82.6 ± 5.9*	93.6 ± 7.4	79.1 ± 4.1*
**Bacteriology**					
*S. aureus* 0–6 years	95.5 (86/90)	95.5 (21/22)	100.0 (21/21)	81.8 (9/11)	97.2 (35/36)
*P. aeruginosa* 0–6 years	62.2 (56/90)	68.2 (15/22)	72.7 (16/22)	54.5 (6/11)	54.3 (19/35)
At least once chronic infection	18.9 (17/90)	27.3 (6/22)	31.8 (7/22)	0 (0/11)	11.4 (4/35)
**Treatment received from 0–6 years**					
Inhaled bronchodilators	74.5 (70/94)	31.8 (7/22)**	75 (18/24)*	83.3 (10/12)*	97.2 (35/36)*
Inhaled corticosteroids	74.5 (70/94)	**40.9 (9/22)**	75 (18/24)*	75.0 (9/12)	94.4 (34/36)*/**

*Mean ± SD; % (n/N).*

*TW, transient earlier wheezers; NW, never wheezers; LW, late onset wheezers; PW, persistent wheezers.*

*p < 0.05 vs. NW; **p < 0.05 TW.

*Bold values indicate the significant difference between the two groups p < 0.05.*

#### Impact on Respiratory Function at 6 Years

The prevalence of “current” wheezers at the age of 6 years was 28.8% (21/73). The impact of wheezing in children according to their wheezing profiles or the existence of “current” wheezing at the age of 6 years is summarized in Tables 2, 3.

**TABLE 3:** Consequences of current’ wheezing at the age of 6 years.

Data	Current wheezers	Not current wheezers	*P*
BMI 6 years (*z*-score)	*n* = 16 0.15 ± 0.26	*n* = 48 −0.11 ± 0.15	NS
ppFEV_1_	*n* = 20 **91.5** ± **4.4**	*n* = 45 **100.9** ± **2.4**	**0.047**
ppFVC	*n* = 20 89.9 ± 4.2	*n* = 45 98.6 ± 2.4	NS
ppFEF_25–75_	*n* = 19 77.7 ± 5.6	*n* = 45 85.0 ± 3.6	NS
**Bacteriology**			
*S. aureus* 0–6 years	95 (19/20)	95.8 (46/48)	NS
***P. aeruginosa***			
*P. aeruginosa* at 6 years	70.0 (14/20)	56.3 (27/48)	NS
Chronic infection	5.0 (1/20)	20.8 (10/48)	NS
**Treatment**			
Short-acting beta-2 agonists	**100 (21/21)**	**82.4 (42/51)**	**0.039**
Inhaled corticosteroids	90.5 (19/21)	82.4 (42/51)	NS

*Mean ± SD; % (n/N).*

*Bold values indicate the significant difference between the two groups p < 0.05.*

**FIGURE 4:**
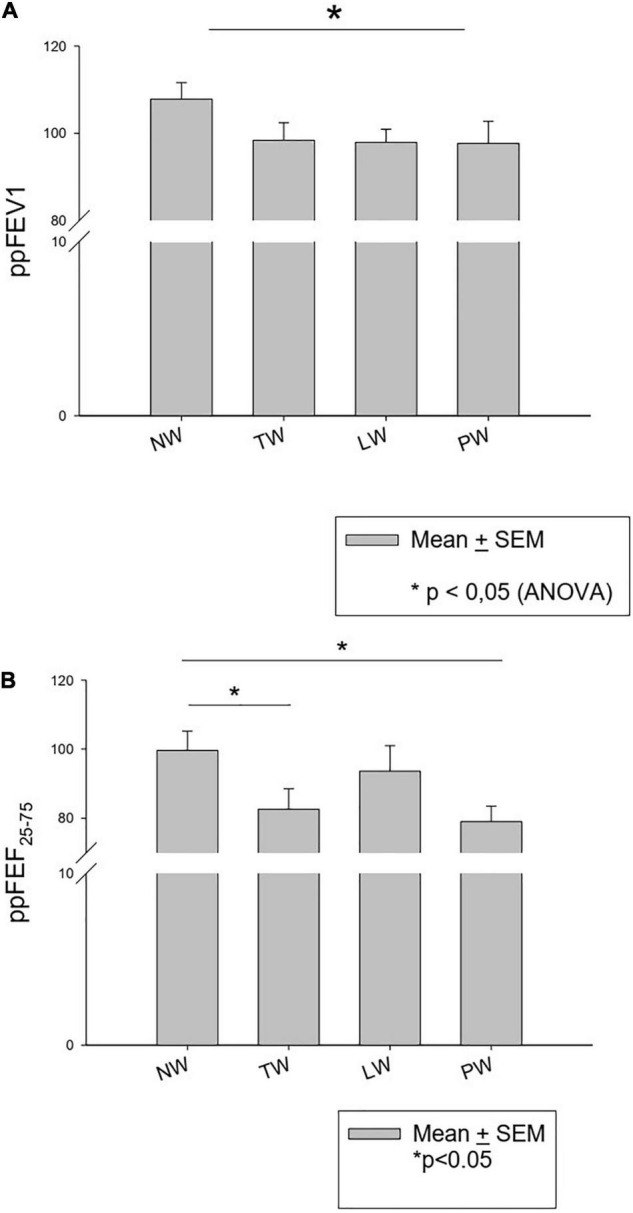
ppFEV_1_ **(A)** and ppFEF_25–75_ **(B)** at the age of 6 years according to the TUCSON wheezing phenotypes.

One of the main consequences of preschool wheezing was its impact on respiratory function. In the present cohort patients who had never wheezed had a mean ppFEV_1_ of 107.8 ± 3.4% vs. 98.0 ± 2.2% in those who had had at least one episode between birth and 6 years of age (*p* = 0.027, Figure 4). Figure 4 shows the ppFEV1 and ppFEF_25–75_ according to the types of wheezing profiles. FEV_1_ and forced vital capacity (FVC) values according to the different TUCSON phenotypes were as follows: NW > TW > PW > LW (NS).

#### Treatments

Prescriptions of short-acting bronchodilators and inhaled corticosteroids were significantly more frequent in symptomatic children (TW < LW < PW) compared to the NW groups (Table 3). At 6 years of age “current” wheezers had received more bronchodilator therapy (*p* = 0.039) and inhaled corticosteroids (NS) vs. non-current wheezers.

### School-Age Children and Teenagers (>6 Years)

#### Demographic Data

Sixty-nine patients were included in this group (Table 4). The M:F sex ratio was 1:1. A Phe508del homozygote genotype was present in 71% (49/69) of cases. Forty-nine children had had at least one annual checkup between ≥6 years and <8 years, 33 children between ≥10 years and <12 years, and 17 children between ≥15 years and <17 years.

**TABLE 4:** Population characteristics of school-aged children (≥6 years) and teenagers.

Data	Total (*N* = 69)
Age (years)	12.2 ± 3.6
Sex ratio (M:F)	34/35
Age at diagnosis (months)	4.3 [0.3–57.8]
**BMI**	
6–8 years	15.5 ± 1.6
10–12 years	16.8 ± 2.2
15–17 years	19.6 ± 2.2
**Genotype**	
DF508 homozygous	49 (71)
Heterozygous	15 (21.7)
Others	5 (7.3)
**ppFEVl**	
6–8 years	89.7 ± 20.4
10–12 years	85.7 ± 15.9
15–17 years	75.2 ± 20.9
**Positive skin prick test (at least one)**	
6–8 years	43/49 (87.8)
10–12 years	29/33 (87.9)
15–17 years	17/17 (100)
***Pseudomonas aeruginosa***	
6–8 years	4/49 (8.2)
10–12 years	7/33 (21.2)
15–17 years	7/17 (41.2)
***Staphylococcus aureus***	
6–8 years	19/47 (40.4)
10–12 years	15/33 (45.5)
15–17 years	11/17 (64.7)
***Haemophilus influenzae***	
6–8 years	7/47 (14.9)
10–12 years	4/33 (12.1)
15–17 years	2/17 (11.7)

*Values expressed as mean ± standard deviation, median [interquartile] or n/N (%).*

#### Prevalence of a Positive Bronchodilatory Response

A positive BDR was found in 73.5, 48.5, and 52.9% of children between 6 and 8 years, 10 and 12 years, and 15 and 17 years, respectively (Table 5). Longitudinally, among the 36 patients with early BDR (at 6–8 years), 8 out of 14 (57%) retained this BDR at 10–12 years, and 3/5 at 15–17 years (Table 5). Among the 13 patients without early BDR criteria, 3 of 6 (50%) progressed to a positive BDR response at the age of 10–12 years.

**TABLE 5:** Prevalence of bronchodilatory responses (BDR).

Profile	6–8 years	10–12 years	15–17 years	*N*
Early BDR	+	+	−	2
	+	+		6
	+	−	+	2
	+	−		4
	+		+	1
	+			21
Absence of early BDR	−	+		3
	−	−	−	1
	−	−		2
	−			7
Other		+	+	1
		+		4
		−	+	1
		−	−	2
		−		5
			+	4
			−	3
Total BDR	36/49	16/33	9/17	69
n/N (%)	(73, 5)	(48, 5)	(52, 9)	

*p = 0.02 (6–8 vs. 10–12 years).*

*BDR according to age (>1 positive lung function BDR criteria, upon annual assessment).*

*+, present; −, absent.*

Using FEV_1_ as a sole criterion, a positive BDR was demonstrated in 34.7, 6.1, and 5.9% of the children between 6 and 8, 10 and 12, and 15 and 17 years of age, respectively (Table 5).

#### Predictive Markers

The homozygous Phe508del genotype was more frequent in the BDR + group compared to the BDR- group (64% vs. 36%, *p* = 0.041) in 6- to 8-year-olds (Table 6). The body mass index in the BDR + group was greater vs. the BDR- group in children aged 10–12 years (*p* = 0.01). The sum of the diameters of the prick test papules for 3 pre-defined common respiratory allergens was greater in the BDR+ patients at all ages, but this was significantly in the 6–8 years age group only (*p* = 0.005). The blood eosinophil counts were significantly lower in the BDR+ group of patients at the age of 10–12 years only (*p* = 0.013). The number of *Aspergillus* precipitation arcs was also lower in the BDR+ group at the age of 10–12 years (*p* = 0.05) (42).

**TABLE 6:** Predictive markers and factors a positive bronchodilatory response (BDR) in patients aged >6 years.

	BDR+ (6–8 years)			BDR+ (10–12 years)			BDR+ (15–17 years)		
	Yes	No	*P*	Yes	No	*P*	Yes	No	*P*
ppFEV_1_	87 ± 20	98 ± 21	NS	87 ± 12	84 ± 19	NS	80 ± 21	70 ± 21	NS
ppFVC	**85** ± **19**	**101** ± **21**	**0.015**	91 ± 10	84 ± 12	NS	83 ± 15	80 ± 16	NS
FEV_1_/FVC	102 ± 9	98 ± 13	NS	96 ± 9	100 ± 14	NS	95 ± 12	86 ± 16	NS
Eos/mm^3^	0.13 [0.11–0.21]	0.20 [0.03–0.46]	NS	0.29 [0.08–0.29]	0.28 [0.21–0.34]	NS	0.23 [0.06–0.43]	0.15 [0.10–0.28]	NS
Eos %	2.35 [1.83–4.00]	2.45 [1.85–5.00]	NS	**2.90 [1.60–3.70]**	**5.20 [2.75–7.40]**	**0.013**	2.20 [0.80–5.23]	1.70 [0.93–2.80]	NS
IgE (kUI/l)	37 [14–132]	20 [3–225]	NS	41 [27–121]	178 [30–670]	NS	35 [21–251]	89 [36–352]	NS
Sum SPT (mm)	**4 [2.0–8.8]**	**1 [0–7]**	**0.012**	4 [2–7.8]	3 [0.5–8.5]	NS	7 [5.5–8.5]	6 [1.5–7.8]	NS
Asp. Arcs (number)	0.47 ± 0.08 0 [0–0]	0.55 ± 0.15 0 [0–0]	NS	**0.0** ± **0.0** 0 [0–0]	**0.9 ± 1.7** 0 [0–1.7]	**0.05**	1.6 ± 2.5 0 [0–3]	0.5 ± 0.7 0 [0–l]	NS
Pa (log CFU/ml)	0.2 ± 0.7 0 [0–0]	0.6 ± 2.1 0 [0–0]	NS	1.2 ± 3.9 0 [0–0]	1.4 ± 2.8 0 [0–1.5]	NS	4.8 ± 7.6 0 [0–7.5]	1.1 ± 1.6 0 [0–2.8]	NS
Sa (log CFU/ml)	2.6 ± 3.4 0 [0–7]	2.5 ± 3.4 0 [0–7]	NS	2.9 ± 3.2 0 [0–6]	3.0 ± 3.9 0 [0–8]	NS	2.7 ± 2.8 3 [0–5.5]	4.0 ± 3.5 4 [0.3–7]	NS
Hi (CFU/ml)	0.7± 1.9 0 [0–0]	0.9 ± 1.9 0 [0–l]	NS	0.4 ± 1.7 0 [0–0]	1.2 ± 2.8 0 [0–0]	NS	0.0 ± 0.0 0 [0–0]	1.8 ± 3.4 0 [0–3.8]	NS
Sum log CFU Pa+Sa+Hi	3.3 ± 4.2 0 [0–7]	3.8 ± 1.0 5 [0–7]	NS	4.6 ± 6.4 2 [0–7.5]	5.6 ± 5.6 6 [0–8.5]	NS	7.4 ± 6.5 6 [4–9]	6.9 ± 5.5 7 [2–8.8]	NS
LABA	14/35 (40)	4/13 (31)	NS	6/16 (37)	8/17 (47)	NS	4/8 (50)	6/8 (75)	NS
Macrolide	18/35 (51)	7/13 (54)	NS	9/16 (56)	12/17 (71)	NS	9/9 (100)	6/8 (75)	NS
Wheezing	–	–	–	–	–	–	–	–	–
Phe508del (Homozygote)	**16/25 (64)**	**9/25 (36)**	**0.041**	11/17 (65)	6/17 (35)	NS	3/7 (43)	4/7 (57)	NS
Body weight (kg)	21.5 ± 4.3	21.9 ± 4.2	NS	36.7 ± 8.9	31.4 ± 7.3	NS	53.2 ± 6.0	50.3 ± 14.5	NS
Height (m)	1.17 ± 0.08	1.18 ± 0.06	NS	1.43 ± 0.11	1.40 ± 0.09	NS	1.64 ± 0.06	1.6 ± 0.12	NS
BMI	15.5 ± 1.6	15.6 ± 1.8	NS	**17.8** ± **2.1**	**15.8** ± **1.9**	**0.01**	19.8 ± 1.5	19.4 ± 3.0	NS

*Mean ± SD; % (n/N).*

*BMI, Body Mass Index; Eos, blood eosinophils; IgE, immunoglobulin type E; SPT, skin pricks tests; Arc Asp., arcs Aspergillus; Pa, Pseudomonas aeruginosa; Sa, Staphylococcus aureus; Hi, Haemophilus influenzae; LABA: long-acting beta 2 agonists.*

*Bold values indicate the significant difference between the two groups p < 0.05.*

#### Impact of a Positive Bronchodilatory Response on Lung Function

FVC and FEV1 values of BDR + patients at the age of 6–8 years were lower than those of BDR- patients, with a significant difference regarding FVC only (85% vs. 101%, *p* = 0.015, Table 6).

## Discussion

The present study indicates that wheezing is a common phenomenon in preschool children with CF. Wheezing was not associated to p.Phe508del homozygosity, allergic factors or chronic *Pseudomonas aeruginosa* colonization. Persistent wheezing was associated with lower lung function at the age of 6 years. A positive BDR in older children with CF was also a common finding, and was associated with lower lung function between 6 and 8 years of age, increased BMI between 10 and 12 years of age, and a greater sum of the diameter of allergic prick test papules for three common respiratory allergens. In this study, we did not find identify allergic bronchopulmonary aspergillosis as a cause of wheezing.

The reported rate of wheezing in CF is variable. In a large population of patients with CF in the United States and Canada, the cumulative percentage of patients reaching onset of persistent wheezing was approximately 30% at the age of 6 years and 50% at the age of 15 years (17). In contrast, the European CF Epidemiologic Registry (ERCF) reported a rate of asthma of 14% in children with CF less than 6 years of age. Ren et al. showed that wheezing during the first 6 years of life was associated with lower lung function at the age of 6–8 years (18, 19). Based on the different TUCSON phenotypes (16), they reported that persistent wheezers (PW) had significantly lower FEV1 than never wheezers (NW) (19). Levine et al. showed that 39% of children aged 14.4 (4–76) years, median (range), with CF had reversible bronchial obstruction (20).

Physicians tend to extrapolate asthma treatment to the management of wheezing in CF. We and others (1, 17, 20) raise concerns regarding the abuse of inhaled steroids and bronchodilators which are too often prescribed in patients with CF. According to the French 2019 CF registry, 36.3 and 59.3% of patients received inhaled corticosteroids and bronchodilators, respectively (21). Our work confirmed the widespread use of these agents: short-acting bronchodilators were prescribed in 97.2% of PWs and 94.4% of such wheezers received inhaled corticosteroids. Ren et al. reported that 89.1% of PW received bronchodilators vs. 73.2% of NW (*p* < 0.05). Kent et al. (22) pointed out that there was little evidence for the efficacy of asthma therapy in CF. A Cochrane review concluded that no study has been able to demonstrate that the use of inhaled corticosteroids decreases lung inflammation in CF (23). In France, a Delphi study recommended restricting the use of inhaled corticosteroids in CF due to an insufficient level of evidence (24). According to Smith and Edwards neither long-acting beta-2 agonists nor long-acting muscarinic antagonist bronchodilators demonstrate improvement in FEV1 in CF (25). Moreover, the side effects of inhaled corticosteroids are not negligible. They can cause oral fungal infections and at high doses may negatively impact growth. Overall, under close supervision, teams caring for patients with CF may safely stop prescribing inhaled corticosteroids in these patients (26).

### Preschool Children (<6 Years)

Wheezing in children with CF is more frequent compared to non-CF children [33.6% at 3 years of age and 48.6% at 6 years of age in the TUCSON Cohort (16)]. McColley et al. (17) found that children diagnosed by neonatal screening had a reduced risk of early onset of wheezing. The early diagnosis of the disease (associated with appropriate overall management by pediatric expert centers) is of vital importance to preserve the respiratory status in CF (14).

#### Predictive Markers

In our study, a positive API between 0 and 3 years of age was not significantly associated with any TUCSON wheezing phenotype, or with “current” wheezing at the age of 6 years. The PIAMA score was also not contributory. Personal allergic diseases (eczema, allergic rhinitis, etc.) were relatively uncommon and non-discriminatory. There was a 2 to threefold lower prevalence of eczema in the NW than in the 3 other subgroups (NS). No other criteria (birth weight, gestational age, parental asthma, allergic SPT, and smoking) was significantly associated with a particular TUCSON wheezing profile. The “protective” role of p.Phe508del homozygote mutations representing 72.7% of the children in the NW group compared to 32% in the TW group (*p* = 0.009) and 41.7% in the PW group (*p* = 0.037) was not found by Ren et al. (19). Chronic colonization with *P. aeruginosa* at 3 years of age was not associated with “current” wheezing at 6 years of age (*p* = 0.044). Interestingly, McColley et al. (17) found that the presence of *P. aeruginosa* and/or *Staphylococcus aureus* seemed to be “protective” for the occurrence of crackles and chest congestion. They suggested that the treatments and more stringent follow-up when these bacteria were present may have played a “protective” role.

#### Consequences of Preschool Wheezing in Cystic Fibrosis

In our study, ppFEF_25–75_ was significantly greater in NW compared to PW (*p* = 0.027). Ren et al. also reported higher FEV_1_ and FVC values in NW compared to the other phenotypes (19). Preschool wheezing had a significant impact on lung function at school age in our study. In current wheezers at the age of 6 years ppFEV_1_ was impaired [91.5% vs. 100.9% in non-wheezers since 12 months (*p* = 0.047)], as shown in previous reports (16, 19). Preschool wheezing did not significantly affect Body Mass Index at 6 years of age even though “current” wheezers had a −0.52 lower BMI z-score compared to the “non-current” wheezers, or the microbiological status in the sputum.

### School-Age Children and Teenagers (≥6 Years)

We did not specifically study the prevalence of wheezing in school-age children. However, we did demonstrate that BDR was a common phenomenon involving approximately half of the patients studied. Mitchell et al. reported BHR after methacholine challenge in 51% of children with CF, compared to 98% in asthmatic control children (7). Mellis and Levison identified 24% of histamine responders among CF patients, compared to 90% in asthmatic control patients (27). In addition, there appears to be two categories of CF patients: those with symptoms of asthmatic disease, clinically diagnosed on the basis of recurrent acute exacerbations of wheezing, with a very high efficacy of bronchodilators and/or systemic corticosteroids; and those with some degree of “isolated” BHR (non-asthmatics) (9, 47).

Bronchial responsiveness to bronchodilators is an integrated physiological response involving airway epithelium, nerves, mediators and bronchial smooth muscle. In 1993, an *ad hoc* ATS committee recommended that the diagnosis of asthma be based either on methacholine/histamine challenges, or repeat spirometry after beta-adrenergic agonists or steroid trials (5). This suggests that bronchoconstrictor responsiveness (BCR) and BDR may be considered physiological opposites in chronic obstructive airways disease. In many LFT laboratories, provocation tests have been replaced by bronchodilator tests in the assessment of cases of airways obstruction. However, the correlation between bronchoconstriction and bronchodilator response is imperfect and it is not possible to infer with certainty the presence of one from the other (28). In COPD symptoms were more associated with the presence of a BCR, but not a BDR, indicating that they are two different phenotypic markers that are not interchangeable (29).

In the present study, a greater sum of allergic SPT HDM + Cat + Dog papules, lower lung function values and homozygous Phe508del genotypes were associated with a positive BDR in 6–8 year-old children. BDR+ patients aged 10–12 had lower blood eosinophil counts and *Aspergillus* precipitin arcs, as well as a higher body mass index, compared with BDR-children. In a similar study, Levin et al. showed that reversible obstruction (ΔppFEV1 ≥ 12%) was associated with younger age (*p* = 0.01) and a severe genotype (*p* = 0.02), but not with a family history of asthma, serum IgE, blood eosinophils, pancreatic status, ppFEV1 < 40%, *Aspergillus* or *Pseudomonas* infection (20). In a study by Van Haren et al. including 20 children with CF, 40% of patients had histamine BHR, mainly among the youngest, and this was correlated with positive allergic SPTs (30). In children with CF, the prevalence of positive markers of respiratory allergy is significantly higher than in the general population (9). In a study including 31 adult patients with CF, such sensitization was found in 65% of patients with BHR (31). In three other studies performed in adults after challenge testing, BHR was also not correlated with allergic SPT positivity (7, 27, 32). Our study allowed us to observe the BDR with a longitudinal perspective. Regardless of the criteria used, the prevalence of a BDR was greater in the youngest patients and had decreased by the age of 10–12 years. Overall, allergy appears to be inconsistently associated with BHR and BDR + in CF.

#### Mechanisms of Wheezing and Bronchial Hyperresponsiveness in Cystic Fibrosis

According to McCuaig and Martin, deficient ion transport across CFTR in patients with CF cannot be solely responsible for the altered ASM physiology, as there is as much smooth muscle hypertrophy in pediatric CF patients as in those with non-CF bronchiectasis (10). Both CF and non-CF bronchiectasis are diseases characterized by high levels of neutrophils in the lungs, suggesting an important contribution of the inflammatory environment to ASM alterations. The presence of a bacterial infection, particularly *Pseudomonas*, will promote the secretion of IL-8 and TNF-alpha, leading to bronchial remodeling. In addition, CFTR-deficient T cells will be directed to type 2 T-helper cells, which will cause a pro-allergic response (10). In addition, calcium responses are altered in CFTR-deficient ASM at a very early stage, as confirmed by studies in CFTR−/− neonatal pigs. Ca^++^ is a crucial second messenger in smooth muscle contraction, activating myosin light chain (MLC) kinase through the formation of a Ca^++^ and calmodulin complex. The release of Ca^++^ and the activation of Cl- channels in the sarcolemma of ASM may be of importance in the smooth muscle contraction in CF, in contrast to asthma (33). Other possible causes of wheezing include antenatal cigarette smoke exposure (16), small or floppy airways (34, 35), excess intraluminal mucus (34), gastro-intestinal reflux (36), and oxidant-antioxidant species imbalance (37). Gastro-esophageal reflux, one of the most common gastrointestinal manifestations of CF, probably plays a role in the pathogenesis of the airway disease by inducing repeated micro-aspiration and bronchospasm (36). An altered redox environment with a low concentration of antioxidants, in particular glutathione, contrasting with high levels of 8-isoprostane in the epithelial surface liquid contributes to progressive lung damage (37).

#### Study Limitations

This study was retrospective and monocentric. This explains the small number of patients compared to other similar studies. However, missing data were limited since we have used prospective standardized national CF Registry procedures and dedicated software (MucoDomeos).^1^

***Overall***, based on the different data from the literature and from our own study the following hypothesis can be proposed: most children with CF behave like non-specific asthmatic patients, with a high prevalence of BHR and/or response to bronchodilators. They would then evolve toward a form of chronic obstructive airway disease with a lower prevalence of BHR. This raises the question as to which are the most appropriate treatments of BHR in CF. At the present time, the most promising agents are CFTR modulators. VX-809/770, reduces the ASM cell proliferation and normalizes calcium reuptake kinetics (38); Ivacaftor rapidly improves airflow obstruction, air trapping and airway distensibility (39); Lumacaftor-ivacaftor improves LCI (−1.6, 95% CI −2.6 to −0.5; *P* < 0.01), airway microbiota and inflammation, as well as MRI morphology (−1.3, 95% CI −2.3 to −0.3; *P* < 0.05) and perfusion score (−1.2, 95% CI −2.3 to −0.2; *P* < 0.05) (40). Ribeiro and Gentzsch have suggested that CF airway epithelial inflammation may enhance the efficacy of CFTR modulators, and this could have clinical implications regarding the presence of wheezing (41).

## Conclusion

While wheezing is very common in children with CF, no major determinants in children below 6 years of age could be clearly identified. Current wheezing at age 6 years was significantly associated with lower lung function. In children older than 6 years, allergic factors, genetics (p.F508del homozygosity) and higher BMI were significantly associated with the presence of a positive BDR but this varied between age groups. In 6–8 years old children with a BDR, baseline lung function was significantly lower. Our results suggest that the “CF-Asthma” designation may be misleading in a vast majority of cases, and may lead to inappropriate treatment with inhaled steroids, especially in preschool wheezers.

## Data Availability Statement

The original contributions presented in the study are included in the article/supplementary material, further inquiries can be directed to the corresponding author/s.

## Ethics Statement

Ethical approval was not provided for this study on human participants because not yet, processing. Written informed consent to participate in this study was provided by the participants’ legal guardian/next of kin.

## Author Contributions

All authors listed have made a substantial, direct, and intellectual contribution to the work, and approved it for publication.

## Conflict of Interest

MF is affiliated (but not employed) by the INSERM, CIC 1401. The remaining authors declare that the research was conducted in the absence of any commercial or financial relationships that could be construed as a potential conflict of interest.

## Publisher’s Note

All claims expressed in this article are solely those of the authors and do not necessarily represent those of their affiliated organizations, or those of the publisher, the editors and the reviewers. Any product that may be evaluated in this article, or claim that may be made by its manufacturer, is not guaranteed or endorsed by the publisher.

## Footnotes

1
https://www.vaincrelamuco.org/2019/05/09/mucodomeos-un-logiciel-adapte-aux-besoins-des-crcm-2684

## Abbreviations

ASM: airway smooth muscle
BDR: bronchodilatory response
BHR: bronchial hyperresponsiveness
IgE: type E immunoglobulin
API: Asthma Predictive Index
IS: induced sputum
LW: late (onset) wheezers
NW: never wheezers
PW: persistent wheezers
PIAMA: prevention and incidence of asthma and mite allergy
SPT: skin prick tests
TW: transient (earlier) wheezers.

## References

[1] Armstrong DS Hook SM Jamsen KM Nixon GM Carzino R Carlin JB Lower airway inflammation in infants with cystic fibrosis detected by newborn screening. *Pediatr Pulmonol.* (2005) 40:500–10. 10.1002/ppul.20294 16208679

[2] Bush A Tiddens H Silverman M. Clinical implications of inflammation in young children. *Am J Respir Crit Care Med.* (2000) 162:S11–4. 10.1164/ajrccm.162.supplement_1.maic-3 10934124

[3] Hays SR Ferrando RE Carter R Wong HH Woodruff PG. Structural changes to airway smooth muscle in cystic fibrosis. *Thorax.* (2005) 60:226–8. 10.1136/thx.2004.028340 15741440 PMC1747321

[4] Regamey N Ochs M Hilliard TN Muhlfeld C Cornish N Fleming L Increased airway smooth muscle mass in children with asthma, cystic fibrosis, and non-cystic fibrosis bronchiectasis. *Am J Respir Crit Care Med.* (2008) 177:837–43. 10.1164/rccm.200707-977OC 18218992

[5] Miller A. Guidelines for the evaluation of impairment/disability in patients with asthma. *Am J Respir Crit Care Med.* (1994) 149(3 Pt 1):834–5. 10.1164/ajrccm.149.3.8155167 8155167

[6] Burdon JG Cade JF Sutherland PW Pain MC. Cystic fibrosis and bronchial hyperreactivity. Concomitant defects or cause and effect ? *Med J Aust.* (1980) 2:77–8. 10.5694/j.1326-5377.1980.tb76884.x 7421655

[7] Mitchell I Corey M Woenne R Krastins IR Levison H. Bronchial hyperreactivity in cystic fibrosis and asthma. *J Pediatr.* (1978) 93:744–8. 10.1016/s0022-3476(78)81070-1 712474

[8] Tobin MJ Maguire O Reen D Tempany E Fitzgerald MX. Atopy and bronchial reactivity in older patients with cystic fibrosis. *Thorax.* (1980) 35:807–13. 10.1136/thx.35.11.807 7221975 PMC471388

[9] Weinberger M. Airways reactivity in patients with CF. *Clin Rev Allergy Immunol.* (2002) 23:77–85. 10.1385/CRIAI:23:1:077 12162108

[10] McCuaig S Martin JG How the airway smooth muscle in cystic fibrosis reacts in proinflammatory conditions: implications for airway hyper-responsiveness and asthma in cystic fibrosis. *Lancet Respir Med.* (2013) 1:137–47. 10.1016/S2213-2600(12)70058-9 24429094

[11] Caudri D Wijga A Maarten C Hoekstra M Postma DS Koppelman GH Predicting the long-term prognosis of children with symptoms suggestive of asthma at preschool age. *J Allergy Clin Immunol.* (2009) 124:903–10. 10.1016/j.jaci.2009.06.045 19665765

[12] Hafkamp-de Groen E Lingsma HF Caudri D Levie D Wijga A Koppelman GH Predicting asthma in preschool children with asthma-like symptoms: validating and updating the PIAMA risk score. *J Allergy Clin Immunol.* (2013) 132:1303–10. 10.1016/j.jaci.2013.07.007 23987795

[13] Castro-Rodriguez JA Holberg CJ Wright AL Martinez FD. A clinical index to define risk of asthma in young children with recurrent wheezing. *Am J Respir Crit Care Med.* (2000) 162:1403–6. 10.1164/ajrccm.162.4.9912111 11029352

[14] Burgel PR. Evolutions épidémiologiques de la mucoviscidose en France: perspectives à 10 ans. *Arch Pediatr.* (2016) 23:S4–12. 10.1016/S0929-693X(17)30056-8 28231893

[15] Toulouse E Masseguin C Lafont B McGurk G Harbonn A Roberts JA French legal approach to clinical research. *Anaesth Crit Care Pain Med.* (2018) 37:607–14. 10.1016/j.accpm.2018.10.013 30580775

[16] Martinez FD Wright AL Taussig LM Holberg CJ Halonen M Morgan WJ. Asthma and wheezing in the first six years of life. The group health medical associates. *N Engl J Med.* (1995) 332:133–8. 10.1056/NEJM199501193320301 7800004

[17] McColley SA Ren CL Schechter MS Regelmann WE Pasta DJ Konstan MW. Risk factors for onset of persistent respiratory symptoms in children with cystic fibrosis. *Pediatr Pulmonol.* (2012) 47:966–72. 10.1002/ppul.22519 22359344 PMC4182956

[18] Balfour-Lynn IM Elborn JS. “CF asthma”: what is it and what do we do about it? *Thorax.* (2002) 57:742–8. 10.1136/thorax.57.8.742 12149539 PMC1746393

[19] Ren CL Konstan MW Rosenfeld M Pasta DJ Millar SJ Morgan WJ Early childhood wheezing is associated with lower lung function in cystic fibrosis. *Pediatr Pulmonol.* (2014) 49:745–50. 10.1002/ppul.22894 24123917 PMC4107871

[20] Levine H Cohen-Cymberknoh M Klein N Hoshen M Mussaffi H Stafler P Reversible airway obstruction in cystic fibrosis: common, but not associated with characteristics of asthma. *J Cyst Fibros.* (2016) 15:652–9. 10.1016/j.jcf.2016.01.003 26826913

[21] Vaincrelamuco. *Registre de la Mucoviscidose.* (2019). Available online at: https://www.vaincrelamuco.org/sites/default/files/registre_2019_vf.pdf.

[22] Kent BD Lane SJ Van Beek EJ Dodd JD Costello RW Tiddens HA. Asthma and cystic fibrosis: a tangled web. *Pediatr Pulmonol.* (2014) 49:205–13. 10.1002/ppul.22934 24420817

[23] Balfour-Lynn I. Asthma in cystic fibrosis. *J R Soc Med.* (2003) 96 (Suppl. 43):30–4. PMC1308785 12906323

[24] Fayon M Corvol H Chiron R Bui S. le groupe de travail inflammation de la société Française de la mucoviscidose. Consensus national sur les modalités de prescription des corticoïdes inhalés dans la mucoviscidose [National consensus regarding the prescription of inhaled corticosteroids in cystic fibrosis]. *Arch Pediatr.* (2014) 21:88–94. 10.1016/j.arcped.2013.10.016 24309202

[25] Smith S Edwards CT. Long-acting inhaled bronchodilators for cystic fibrosis. *Cochrane Database Syst Rev.* (2017) 12:CD012102. 10.1002/14651858.CD012102 PMC6486268 29253920

[26] Balfour-Lynn IM Welch K Smith S. Inhaled corticosteroids for cystic fibrosis. *Cochrane Database Syst Rev.* (2019) 7:CD001915. 10.1002/14651858.CD001915.pub6 PMC6609325 31271656

[27] Mellis CM Levison H. Bronchial reactivity in cystic fibrosis. *Pediatrics.* (1978) 61:446–50. 643418

[28] Pellegrino R Viegi G Brusasco V Crapo RO Burgos F Casaburi R Interpretative strategies for lung function tests. *Eur Respir J.* (2005) 26:948–68. 16264058 10.1183/09031936.05.00035205

[29] Douma WR de Gooijer A Rijcken B Schouten JP Koëter GH Weiss ST Lack of correlation between bronchoconstrictor response and bronchodilator response in a population-based study. *Eur Respir J.* (1997) 10:2772–7. 10.1183/09031936.97.10122772 9493659

[30] Van Asperen PP Manglick P Allen H. Mechanisms of bronchial hyperreactivity in cystic fibrosis. *Pediatr Pulmonol.* (1988) 5:139–44. 10.1002/ppul.1950050304 2973571

[31] Caudri D Savenije OE Smit HA Postma DS Koppelman GH Wijga AH Perinatal risk factors for wheezing phenotypes in the first 8 years of life. *Clin Exp Allergy.* (2013) 43:1395–405. 10.1111/cea.12173 24261948

[32] Van Haren EH Lammers JW Festen J van Herwaarden CL. Bronchial vagal tone and responsiveness to histamine, exercise and bronchodilators in adult patients with cystic fibrosis. *Eur Respir J.* (1992) 5:1083–8. 1426217

[33] Michoud MC Robert R Hassan M Moynihan B Haston C Govindaraju V Role of the cystic fibrosis transmembrane conductance channel in human airway smooth muscle. *Am J Respir Cell Mol Biol.* (2009) 40:217–22. 10.1165/rcmb.2006-0444OC 18757309

[34] Meyerholz DK Stoltz DA Namati E Ramachandran S Pezzulo AA Smith AR Loss of cystic fibrosis transmembrane conductance regulator function produces abnormalities in tracheal development in neonatal pigs and young children. *Am J Respir Crit Care Med.* (2010) 182:1251–61. 10.1164/rccm.201004-0643OC 20622026 PMC3001264

[35] McDermott S Barry SC Judge EP Collins S de Jong PA Tiddens HA Tracheomalacia in adults with cystic fibrosis: determination of prevalence and severity with dynamic cine CT. Radiology. 2009 Aug;252(2):577-86. *Erratum Radiol.* (2015) 275:934. 10.1148/radiol.2522081956 19508990

[36] Bongiovanni A Manti S Parisi GF Papale M Mulè E Rotolo N Focus on gastroesophageal reflux disease in patients with cystic fibrosis. *World J Gastroenterol.* (2020) 26:6322–34. 10.3748/wjg.v26.i41.6322 33244195 PMC7656210

[37] Spicuzza L Parisi GF Tardino L Ciancio N Nenna R Midulla F Exhaled markers of antioxidant activity and oxidative stress in stable cystic fibrosis patients with moderate lung disease. *J Breath Res.* (2018) 12:026010. 10.1088/1752-7163/aa9b39 29146889

[38] Jang JH Panariti A O’Sullivan MJ Pyrch M Wong C Lauzon AM Characterization of cystic fibrosis airway smooth muscle cell proliferative and contractile activities. *Am J Physiol Lung Cell Mol Physiol.* (2019) 317:L690–701. 10.1152/ajplung.00090.2019 31508974

[39] Adam RJ Hisert KB Dodd JD Grogan B Launspach JL Barnes JK Acute administration of ivacaftor to people with cystic fibrosis and a G551D-CFTR mutation reveals smooth muscle abnormalities. *JCI Insight.* (2016) 1:e86183. 10.1172/jci.insight.86183 27158673 PMC4855508

[40] Graeber SY Vitzthum C Pallenberg ST Naehrlich L Stahl M Rohrbach A Effects of Elexacaftor/Tezacaftor/Ivacaftor therapy on CFTR function in patients with cystic fibrosis and one or two F508del alleles. *Am J Respir Crit Care Med.* (2022) 205:540–9. 34936849 10.1164/rccm.202110-2249OC

[41] Ribeiro CMP Gentzsch M. Impact of airway inflammation on the efficacy of CFTR modulators. *Cells.* (2021) 10:3260. 10.3390/cells10113260 34831482 PMC8619863

[42] Rodriguez-Martinez CE Sossa-Briceno MP Castro-Rodriguez JA. Factors predicting persistence of early wheezing through childhood and adolescence: a systematic review of the literature. *J Asthma Allergy.* (2017) 10:83–98. 10.2147/JAA.S128319 28392707 PMC5376126

[43] Midodzi WK Rowe BH Majaesic CM Saunders LD Senthilselvan A. Predictors for wheezing phenotypes in the first decade of life. *Respirology.* (2008) 13:537–45. 10.1111/j.1440-1843.2008.01284.x 18410257

[44] Sagel SD Gibson RL Emerson J McNamara S Burns JL Wagener JS Impact of *Pseudomonas* and *Staphylococcus* infection on inflammation and clinical status in young children with cystic fibrosis. *J Pediatr.* (2009) 154:183–8. 10.1016/j.jpeds.2008.08.001 18822427 PMC2654617

[45] Colombo JL. Long-acting bronchodilators in cystic fibrosis. *Curr Opin Pulm Med.* (2003) 9:504–8. 10.1097/00063198-200311000-00010 14534403

[46] Donohue JF van Noord JA Bateman ED Langley SJ Lee A Witek TJ Jr A 6-month, placebo-controlled study comparing lung function and health status changes in COPD patients treated with tiotropium or salmeterol. *Chest.* (2002) 122:47–55. 10.1378/chest.122.1.47 12114338

[47] Keam SJ Keating GM. Tiotropium bromide. A review of its use as maintenance therapy in patients with COPD. *Treat Respir Med.* (2004) 3:247–68. 10.2165/00151829-200403040-00005 15350163

